# The Inflammatory Signature of RHI in Athletes at Risk of CTE

**DOI:** 10.1002/alz70856_096745

**Published:** 2025-12-24

**Authors:** Carmela Tartaglia, Charles Tator, Lian Lopes Troncoso, Chloe Anastassiadis, Simrika Thapa, Mozhgan Khodadadi

**Affiliations:** ^1^ Tanz Centre for Research in Neurodegenerative Disease, University of Toronto, Toronto, ON, Canada; ^2^ Krembil Brain Institute, University Health Network, Toronto, ON, Canada; ^3^ Canadian Concussion Centre, Krembil Brain Institute, University Health Network, Toronto, ON, Canada; ^4^ Canadian Concussion Centre, Toronto Western Hospital, Krembil Brain Institute, University Health Network, Toronto, ON, Canada; ^5^ Tanz Centre for Research in Neurodegenerative Diseases, University of Toronto, Toronto, ON, Canada; ^6^ Canadian Concussion Centre, Toronto Western Hospital, University Health Network, Toronto, ON, Canada

## Abstract

**Background:**

There is accumulating evidence that repetitive head impact (RHI) in former contact sports athletes is a risk factor for developing chronic traumatic encephalopathy (CTE) and other neurodegenerative diseases. The pathological link between RHI and neurodegeneration remains unknown. There is increasing evidence that inflammation is one of the physiological responses to RHI^1,2^. Previous studies have demonstrated elevated inflammatory cytokines in blood immediately and years after a concussion^2,3^. The aim of this study was to compare the inflammatory profiles between former professional contact sports athletes with RHI (ExPro) and patients with Alzheimer's disease (AD) and Healthy controls (HC).

**Method:**

We used a multiplex proximity extension assay (PEA) technology using Olink Explore Inflammation I and II panel to quantify 737 inflammatory proteins in the cerebrospinal fluid (CSF) of 16 ExPro (mean age=60.9+/‐11), 24 AD (mean age 69.1+/‐9), and 6 HC (mean age=57.0+/‐11). We also evaluated CSF Neurofilament Light (NfL) using Single Molecule Array (SIMOA). An ANCOVA (covariate age) was used to compare inflammatory markers between groups and Pearson correlation was used to evaluate relationship of inflammatory markers with NfL.

**Result:**

There were 87 neuroinflammatory proteins that were significantly different between ExPro, AD and HC. These markers span immune activation, oxidative stress, vascular inflammation, extracellular matrix remodelling, and neuroinflammation. SCRN1 and MDH1 remained significant after FDR correction. SCRN1 is predominantly involved in immune regulation, vesicular trafficking, and stress response while MDH1 is central to metabolic pathways (TCA cycle, redox balance) and plays a role in oxidative stress and metabolic reprogramming. In the ExPro and AD, NfL was correlated with 105 inflammatory markers but after FDR correction was positively correlated with TBCA, LAIR1, KMT1A/CKMT1B, MILR1, ADAMTS1 and GLOD4. These markers play important roles in inflammation, immune regulation, metabolism, and tissue homeostasis.

**Conclusion:**

Our results provide evidence that distinct inflammatory profiles exist between former contact sports athletes with RHI, patients with AD and HC. As well, we show a relationship between markers of inflammation and neurodegeneration. Identifying specific inflammatory markers relevant to RHI could support the development of targeted therapies to halt neurodegeneration in RHI.

1. Neurology 93(5), e497(2019); 2. Front Neurol 4, 18(2013); 3. Concussion 2(1), CNC30(2017).

